# Systematic Analysis of *Cis*-Elements in Unstable mRNAs Demonstrates that CUGBP1 Is a Key Regulator of mRNA Decay in Muscle Cells

**DOI:** 10.1371/journal.pone.0011201

**Published:** 2010-06-21

**Authors:** Jerome E. Lee, Ju Youn Lee, Jeffrey Wilusz, Bin Tian, Carol J. Wilusz

**Affiliations:** 1 Program in Cell and Molecular Biology, Colorado State University, Fort Collins, Colorado, United States of America; 2 Department of Microbiology, Immunology and Pathology, Colorado State University, Fort Collins, Colorado, United States of America; 3 Department of Biochemistry and Molecular Biology, New Jersey Medical School, University of Medicine and Dentistry of New Jersey, Newark, New Jersey, United States of America; Emory University, United States of America

## Abstract

**Background:**

Dramatic changes in gene expression occur in response to extracellular stimuli and during differentiation. Although transcriptional effects are important, alterations in mRNA decay also play a major role in achieving rapid and massive changes in mRNA abundance. Moreover, just as transcription factor activity varies between different cell types, the factors influencing mRNA decay are also cell-type specific.

**Principal Findings:**

We have established the rates of decay for over 7000 transcripts expressed in mouse C2C12 myoblasts. We found that GU-rich (GRE) and AU-rich (ARE) elements are over-represented in the 3′UTRs of short-lived mRNAs and that these mRNAs tend to encode factors involved in cell cycle and transcription regulation. Stabilizing elements were also identified. By comparing mRNA decay rates in C2C12 cells with those previously measured for pluripotent and differentiating embryonic stem (ES) cells, we identified several groups of transcripts that exhibit cell-type specific decay rates. Further, whereas in C2C12 cells the impact of GREs on mRNA decay appears to be greater than that of AREs, AREs are more significant in ES cells, supporting the idea that *cis* elements make a cell-specific contribution to mRNA stability. GREs are recognized by CUGBP1, an RNA-binding protein and instability factor whose function is affected in several neuromuscular diseases. We therefore utilized RNA immunoprecipitation followed by microarray (RIP-Chip) to identify CUGBP1-associated transcripts. These mRNAs also showed dramatic enrichment of GREs in their 3′UTRs and encode proteins linked with cell cycle, and intracellular transport. Interestingly several CUGBP1 substrate mRNAs, including those encoding the myogenic transcription factors *Myod1* and *Myog*, are also bound by the stabilizing factor HuR in C2C12 cells. Finally, we show that several CUGBP1-associated mRNAs containing 3′UTR GREs, including *Myod1*, are stabilized in cells depleted of CUGBP1, consistent with the role of CUGBP1 as a destabilizing factor.

**Conclusions:**

Taken together, our results systematically establish *cis*-acting determinants of mRNA decay rates in C2C12 myoblast cells and demonstrate that CUGBP1 associates with GREs to regulate decay of a wide range of mRNAs including several that are critical for muscle development.

## Introduction

Dramatic changes in cellular gene expression profiles occur in response to extracellular stimuli, and during differentiation [1], [2]. These changes are achieved in part through the actions of transcription factors which allow coordinated regulation of specific gene sets. However, regulation of mRNA stability can facilitate rapid changes in gene expression both independently and in collaboration with transcriptional effects [3]–[5].

Messenger RNA decay rates can be readily modulated by association of specific stabilizing or destabilizing factors with the transcript [6]. These regulatory factors include RNA-binding proteins and/or miRNAs and their associated enzymes. For almost all mRNAs, decay initiates with removal of the poly(A) tail by one or more deadenylases [6]. This has the dual effects of silencing translation and rendering the transcript susceptible to other decay enzymes. It seems that many regulatory RNA binding proteins and miRNAs are able to modulate the efficiency of deadenylation to accelerate or slow down mRNA turnover. CUGBP1, for example, can bind to the 3′UTR of its target mRNAs and recruit the PARN deadenylase to enhance mRNA decay [7]. Binding of HuR, on the other hand, is generally associated with increased mRNA stability [8], [9], but it is not clear whether this is achieved through a direct inhibition of the decay enzymes, or merely by competing for the binding site of instability factors.

Just as transcription profiles vary between cell types, so can mRNA decay rates [10]. Such variations are presumably due to differences in activity of RNA-binding proteins and/or miRNAs that target specific sets of transcripts. We wished to examine rates of mRNA decay in C2C12 muscle cells for three reasons: (i) Previous studies have uncovered changes in mRNA stability that are essential for differentiation in this cell type [11]–[14]. (ii) Recent results from our lab and others have suggested that changes in mRNA decay rates in muscle cells may be responsible for aspects of pathogenesis in myotonic dystrophy (DM) [15], [16]. (iii) Several muscle cell responses require rapid reprogramming of gene expression, including the response to insulin [17], injury [18], membrane depolarization [19] and exercise [20]. Although the overall changes in gene expression have been characterized for many of these responses, the contribution of mRNA decay is unknown. Our goal in the first experiments described here was to establish mRNA decay rates on a genome-wide scale in C2C12 cells, characterize the sequence elements that influence mRNA turnover in this cell type and compare the results with those in other cell types.

CUGBP1 is an RNA-binding protein whose abundance and/or localization is altered in several neuromuscular diseases, including myotonic dystrophy, Fragile X Tremor/Ataxia Syndrome (FXTAS) and Oculopharyngeal Muscular Dystrophy (OPMD). In addition to its well-defined role as a regulator of splicing, CUGBP1 also influences mRNA turnover through association with GU-rich elements (GREs) in the 3′UTR of its target mRNAs [21]. Binding of CUGBP1 to 3′UTR elements results in recruitment of deadenylases such as PARN which can mediate rapid poly(A) shortening. This can induce translational silencing and/or mRNA decay. Thus CUGBP1 is a potent mRNA destabilizing factor. In the later experiments described below, we have determined the full complement of mRNAs associated with CUGBP1 in muscle cells by RNA immunoprecipitation followed by microarray (RIP-Chip).

Overall, we discovered that many unstable mRNAs expressed in muscle contain AU-rich and/or GU-rich elements in their 3′UTRs. AU-rich elements (AREs) are known to influence decay of short-lived mRNAs in many cell types, while the GREs were similar to those recently identified as CUGBP1 binding sites in unstable mRNAs expressed in T-cells [21] and in *Xenopus* [22]. By comparing our results with those recently reported on mRNA decay in pluripotent and differentiating Embryonic Stem (ES) cells [10], we found that GREs are significant in different cell types, whereas some AREs show cell-specific activities. The set of mRNAs associated with CUGBP1 in myoblasts was also enriched for GU-rich 3′UTR sequences, but not AU-rich ones. These CUGBP1-bound mRNAs tend to have short half lives and encode factors involved in processes such as cell cycle regulation, protein localization, signaling, apoptosis and RNA processing. Interestingly, several CUGBP1-associated mRNAs are bound by HuR and/or Pum1 in other cell types suggesting the existence of coordinated or competitive binding of RNA-binding proteins to achieve appropriate regulation. Finally, several CUGBP1 target transcripts were significantly stabilized in a CUGBP1 KD cell line. Taken together, our results strongly implicate CUGBP1 as a key regulator of mRNA decay in muscle cells.

## Results

### Assessment of mRNA decay rates in C2C12 cells

**Figure 1:**
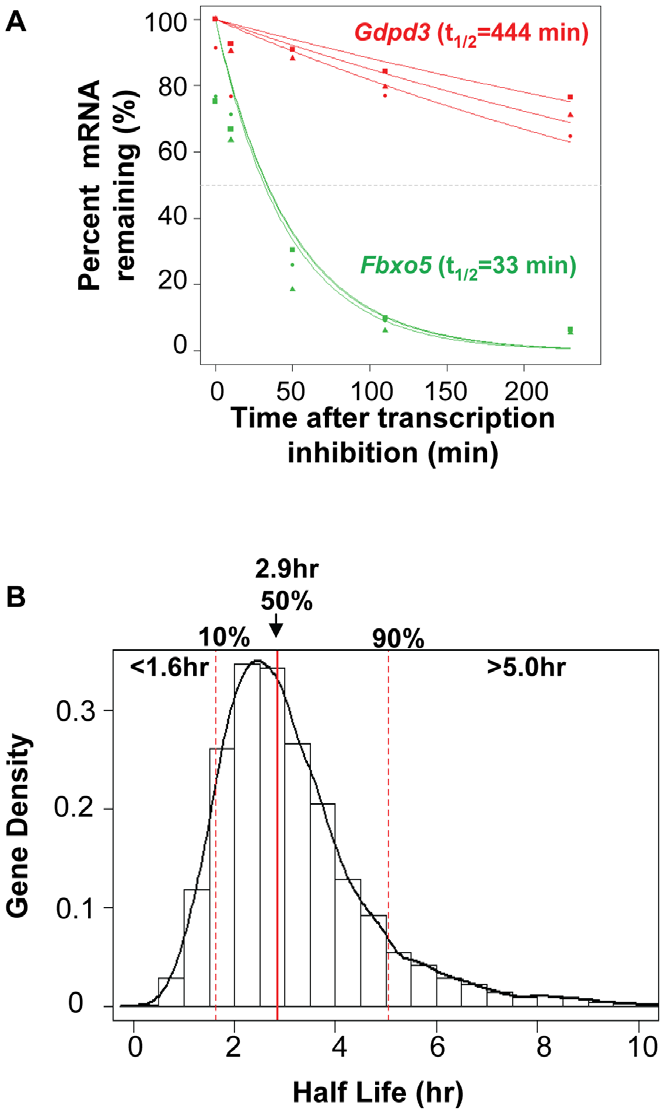
Analysis of mRNA decay rate in C2C12 cells. (**A**) Examples of mRNA decay curves were derived by the nonlinear least squares method for a long and a short half life mRNA (see Materials and Methods for details). (**B**) Distribution of mRNA half lives (see Dataset S1 for the complete list). The 10^th^-percentile and 90^th^-percentile values (indicated by red dotted lines) were used to select mRNAs with short and long half lives, respectively. The median value (2.9 hr) is indicated by a red line.

In order to evaluate mRNA decay rates in muscle cells, we treated C2C12 mouse myoblasts with actinomycin D to inhibit transcription and collected samples at 0, 10, 50, 110 and 230 min. We utilized a relatively short time course to minimize toxic effects of transcription inhibition and to enable more accurate estimation of decay rates for mRNAs with short half lives, as these are more likely to be regulated. Total RNA was prepared from each sample and used to generate DNA probes for hybridization to Affymetrix Mouse Gene 1.0 arrays. The abundance of each mRNA was plotted over time and fitted to a first-order exponential decay curve allowing a half life and confidence interval to be determined (see Materials and Methods for details). The experiment was repeated in triplicate and a mean half life was calculated. Example half lives for two mRNAs, *Gdpd3* and *Fbxo5*, are shown in Figure 1A. In order to identify genes with reliable half life estimates we required that the decay had a good fit (p<0.05) to the exponential curve, and the range of the confidence intervals (95%) for a calculated half life be less than twice the half life. Furthermore, we required that these criteria be met for at least two of the three replicates. In this way we were able to generate reliable half lives for 7398 mRNAs (Dataset S1). The median half life of these transcripts was ∼2.9 hr with 80% of the transcripts decaying with half lives of between 1.6 and 5.0 hrs (Figure 1B). Overall, we find that the half lives we calculated fall within the reported range for mRNAs whose decay in C2C12 cells has been investigated by others. For example, *Myod* mRNA has a half life of ∼93min from our analysis which is consistent with the ∼90 min half life reported [14] and *Igf1* mRNA has a half life of ∼6 hrs similar to that seen previously [23].

### Functional analysis of unstable and stable transcripts

To identify gene functional groups with significantly biased half lives, we examined Gene Ontology (GO) terms for the most and least stable 10% of mRNAs. This revealed that the most unstable mRNAs expressed in myoblasts tend to encode factors with roles in cell cycle, regulation of transcription, establishment and maintenance of chromatin architecture and RNA processing (Table 1). In contrast, the most stable fraction of mRNAs is enriched for transcripts encoding factors involved in ion transport and lipid metabolism (Table 1).

**Table 1:** Top ranked Gene Ontology (GO) terms associated with short or long half life mRNAs in C2C12 cells.

P-value^1^	GO ID, GO Term
**Short half life mRNAs**	
6.30E-07 (56,14)	GO:0007049,cell cycle
6.07E-06 (31,4)	GO:0006325,establishment or maintenance of chromatin architecture
1.19E-05 (55,17)	GO:0006366,transcription from RNA polymerase II promoter
3.49E-05 (51,16)	GO:0006357,regulation of transcription from RNA polymerase II promoter
6.51E-05 (31,6)	GO:0051276,chromosome organization
1.64E-04 (40,12)	GO:0009892,negative regulation of metabolic process
1.64E-04 (40,12)	GO:0031324,negative regulation of cellular metabolic process
2.78E-04 (16,1)	GO:0016071,mRNA metabolic process
3.39E-04 (35,10)	GO:0010629,negative regulation of gene expression
3.39E-04 (35,10)	GO:0045934,negative regulation of nucleobase, nucleoside, nucleotide and nucleic acid metabolic process
4.00E-04 (38,12)	GO:0010605,negative regulation of macromolecule metabolic process
4.08E-04 (18,2)	GO:0048534,hemopoietic or lymphoid organ development
4.83E-04 (29,7)	GO:0045892,negative regulation of transcription, DNA-dependent
4.83E-04 (29,7)	GO:0051253,negative regulation of RNA metabolic process
4.96E-04 (20,3)	GO:0006396,RNA processing
5.56E-04 (24,5)	GO:0000122,negative regulation of transcription from RNA polymerase II promoter
5.56E-04 (24,5)	GO:0000278,mitotic cell cycle
7.39E-04 (17,2)	GO:0006333,chromatin assembly or disassembly
8.45E-04 (33,10)	GO:0016481,negative regulation of transcription
9.87E-04 (14,1)	GO:0006397,mRNA processing
**Long half life mRNAs**	
1.63E-08 (36,4)	GO:0006811,ion transport
1.45E-04 (12,0)	GO:0006820,anion transport
1.63E-04 (20,3)	GO:0006812,cation transport
3.06E-04 (11,0)	GO:0015698,inorganic anion transport
1.35E-03 (9,0)	GO:0015674,di-, tri-valent inorganic cation transport
2.82E-03 (8,0)	GO:0006816,calcium ion transport
2.82E-03 (8,0)	GO:0006817,phosphate transport
2.82E-03 (8,0)	GO:0006887,exocytosis
3.11E-03 (17,4)	GO:0007610,behavior
3.11E-03 (17,4)	GO:0044255,cellular lipid metabolic process
3.17E-03 (15,3)	GO:0030001,metal ion transport
4.68E-03 (10,1)	GO:0006836,neurotransmitter transport
4.68E-03 (10,1)	GO:0007268,synaptic transmission
4.68E-03 (10,1)	GO:0008610,lipid biosynthetic process
5.49E-03 (12,2)	GO:0007626,locomotory behavior
5.90E-03 (7,0)	GO:0006631,fatty acid metabolic process
9.08E-03 (15,4)	GO:0046903,secretion
9.82E-03 (13,3)	GO:0019226,transmission of nerve impulse
9.82E-03 (13,3)	GO:0032940,secretion by cell
1.23E-02 (6,0)	GO:0007601,visual perception

1 P-values were derived from Fisher's exact test, which indicates significance of enrichment of GO terms associated with short half life mRNAs (bottom 10% of all) compared with long half life ones (top 10% of all). The numbers in parenthesis are numbers of mRNAs associated a given GO term in the short and long half-life groups, respectively. Top 20 ranked GO entries are shown for each group.

### GU-rich and AU-rich elements are over-represented in the 3′UTRs of unstable mRNAs

**Figure 2:**
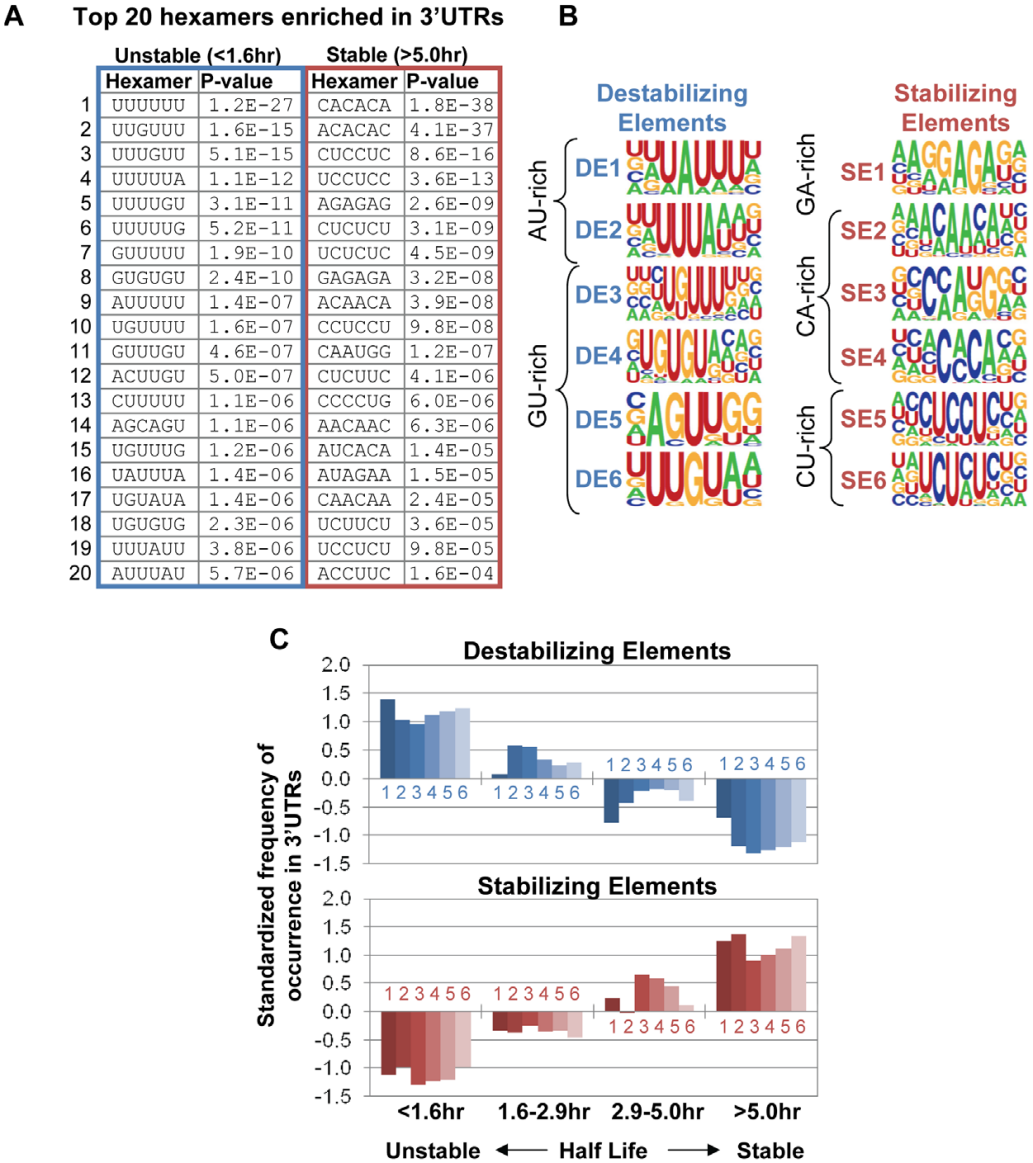
Destabilizing and stabilizing elements in 3′UTRs have combinatorial effects on mRNA stability. (**A**) Top 20 ranked hexamers significantly enriched in the 3′UTRs of mRNAs with short and long half-lives. *P*-values were derived from Fisher's exact test. (**B**) Destabilizing and stabilizing elements (DEs and SEs) were derived by grouping significant hexamers (see Figure S1), and presented as sequence logos. (**C**) DEs and SEs have different frequencies of occurrence in 3′UTRs of mRNAs with different half-lives. mRNAs were divided into 4 groups based on their half life (shown in Figure 1B), i.e. 0–10%, 10–50%, 50–90%, and 90–100%. For each element, the frequencies of occurrence were standardized across the 4 groups by calculating (x-mean)/sd, where x is frequency of occurrence for an element in a group, and mean and sd are mean and standard deviation of frequencies of occurrence for the element in all groups.

We wished to determine whether specific sequence elements are over-represented in stable versus unstable mRNAs. Using the most (t_1/2_>5.0hr) and least (t_1/2_<1.6 hr) stable 10% of transcripts, we examined the 3′UTRs for hexamers that were over-represented in one set as compared to the other. The scores for each possible hexamer are shown in Table S1. The rationale for using hexamers is that many RNA elements are short sequences around six nucleotides [24] and previous studies have shown good selectivity and sensitivity in using hexamers for identifying RNA elements [25], [26]. As shown in Figure 2A, we found that top ranked hexamers include U-rich (>3 consecutive Us), GU-rich, termed GREs, (which contain UGU flanked by Us, UGUG, or GUGU), and AU rich elements or AREs (which contain AUUUA). We then selected all significant hexamers (P<0.01) and grouped them to build consensus *cis*-element motifs and sequence logos (Figure S1). The *cis*-elements enriched in unstable RNAs were termed Destabilizing Elements (DEs; Fig 2B). Notably, *cis*- elements corresponding to GREs (DE3–6) are far more significant than those corresponding to AREs (DE1 and DE2), both in terms of number of significant hexamers and their P-values.

### GA-, CA- and CU-rich elements are enriched in the 3′UTRs of stable mRNAs

The converse analysis revealed that specific sequence elements (Stabilizing Elements, SEs) are also over-represented in the most stable mRNAs (Figures 2A and B). The major elements we identified in the stable mRNAs are GA-rich (SE1), CA-rich (SE2–4) or CU-rich (SE5 and 6). Interestingly, elements related to each of these have been shown previously to have stabilizing effects for individual genes: a GA-rich element has been implicated in stabilizing the elastin mRNA [27], CA repeats bind to hnRNP L and confer resistance to decay [28] and CU-rich and pyrimidine-rich elements interact with HuR or αCP complexes to stabilize mRNAs [29], [30]. To the best of our knowledge, our analysis is the first to establish a stabilizing function for such elements by a systematic approach.

### *Cis*-acting elements act combinatorially to determine mRNA decay rates

Next we examined distributions of each DE and SE in different groups of mRNAs with respect to half life. As we would predict, DEs predominate in unstable mRNAs and SEs in stable mRNAs (Figure 2C). However, a gradual change of occurrence can be discerned for all DEs and SEs as mRNA half lives increase. This result suggests that the overall half life of a given mRNA is combinatorially determined by both DEs and SEs. To further explore this notion, we applied a linear regression model, in which each element was considered as a variable in determining the half life of the mRNA. We built position-specific scoring matrices (PSSM) for all the DEs and SEs and used them to scan mRNA sequences. For completeness, we also scanned 5′UTRs and coding sequences (CDS). Each element received a score for a given region, based on match with the consensus sequence. As shown in Table S2, we found that certain *cis*-elements make larger contributions to the overall mRNA decay rate than others. When located in the 3′UTR ARE (DE2), GRE (DE3), and CA-rich (SE2) sequences are most significant in determining mRNA half life in C2C12 cells. While none of the elements appear to play a role when located in CDS, some, such as DE3 (GRE), and SE4 (CA-rich), may contribute to mRNA decay rate when in the 5′UTR. Importantly, the roles of all significant elements in determination of mRNA half life based on the linear model are in good agreement with our hexamer analysis, i.e. DEs are destabilizing elements and SEs are stabilizing elements. In addition, using this linear model we found that the lengths of 5′UTR and CDS do not significantly influence mRNA decay rates, and the size of 3′UTR is only marginally significant, suggesting specific elements rather than sequence length determine mRNA half life in C2C12 cells.

### *Cis*-element contributions vary between cell types

**Figure 3:**
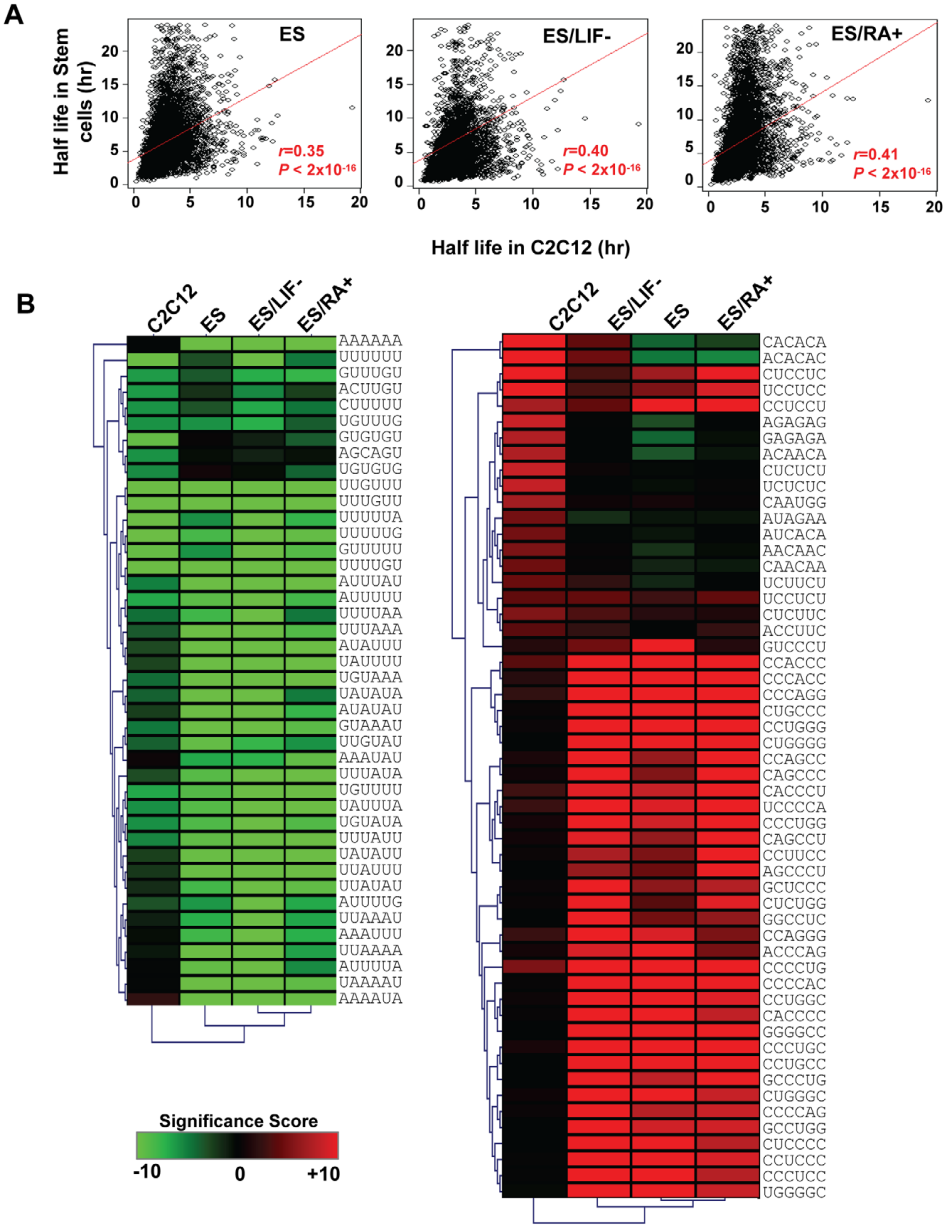
mRNA decay is influenced by different elements in different cell types. (**A**) Scatter plots comparing mRNA half lives in C2C12 with those in pluripotent and differentiating embryonic stem (ES) cells [10]. ES/LIF− and ES/RA+ represent ES cells differentiated by withdrawal of Leukemia Inhibitory Factor (LIF) and addition of Retinoic Acid (RA), respectively. Pearson correlation coefficient (*r*) and *P*-value for linear regression are shown for each plot. (**B**) Heat maps showing significance of hexamers associated with mRNAs with short (left panel) and long (right panel) half lives in C2C12, ES, ES/LIF−, and ES/RA+. Top 20 ranked hexamers for each cell type were selected and combined to illustrate variable significance in different cells. Each hexamer has a Significance Score (SS). SS = −log(*P*-value)*s, where *P*-value was derived from Fisher's exact test, and s = 1 if the hexamer is significantly associated with mRNAs with short half life, and  = −1 otherwise. SS are shown in the heat map with color according to the scale shown in the figure. Hexamers were clustered (Euclidean distance and average linkage) for easy visualization.

To examine whether regulation of mRNA decay rate is cell-specific, we compared our half lives to those generated in a recent analysis of mRNA decay rates in mouse pluripotent and differentiating mouse ES cells [10]. Interestingly, although there is a good overall correlation between the data sets (Figure 3A) there are many mRNAs that exhibit differential decay rates between C2C12 cells and ES cells under the three conditions tested: pluripotent (+Leukemia Inhibitory Factor, LIF), differentiating in the absence of LIF, and differentiating in the presence of retinoic acid (RA) (Figure 3B). It is important to note that this difference is not purely due to technical differences, as the half life discrepancies vary between the three ES cell conditions when each is compared to C2C12 (Figure S2A). Using GO analysis, we found that some gene functional groups are more stable in some cell types than others (Figure S2B). For example, mRNAs associated with ‘translation’ are more stable in pluripotent ES cells than other cell types while those linked with ‘biological adhesion’ have the opposite trend.

We next examined whether specific *cis*-elements impact mRNA half lives more in certain cell types. We applied the same method used for C2C12 cells to the ES cell data. As shown in Figure 3B, while some elements are equally significant in different cell types, some have dramatic differences. Scores for all hexamers are shown in Table S1. Most notable are UGUGUG and GUGUGU which are highly significant in unstable mRNAs in C2C12 but not in other cells, and several general AU rich elements (not AUUUA) are highly significant in ES and differentiating ES cells but not in C2C12. Differences can also be discerned for stabilizing elements (Figure 3B right panel). Thus we conclude *cis*-elements are utilized differently in different cells.

### CUGBP1 associates with GRE-containing mRNAs in myoblasts

**Figure 4:**
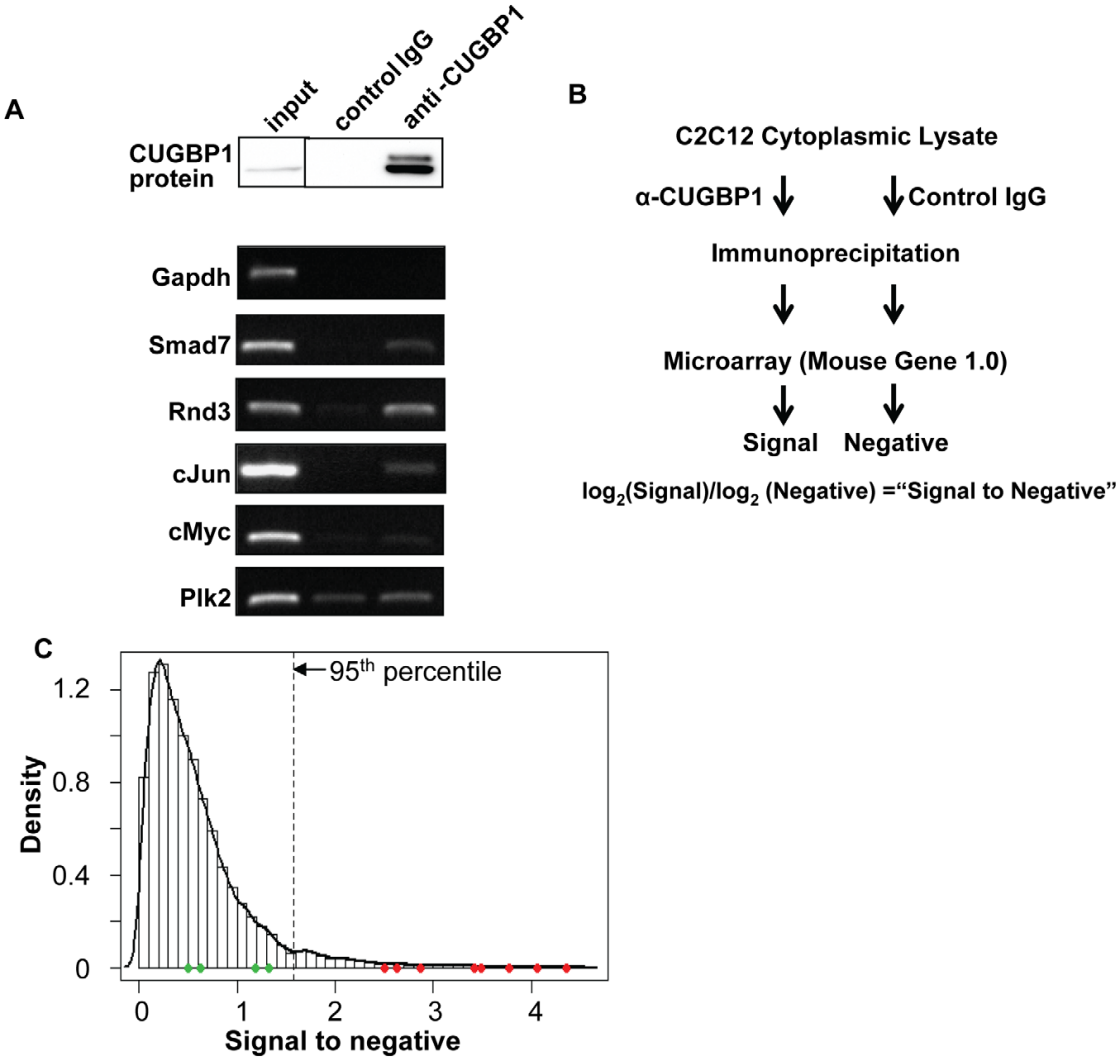
Identification of CUGBP1-associated mRNAs. (**A**) Western blot showing efficient immunoprecipitation of CUGBP1 from cytoplasmic extracts of C2C12 (LKO1) cells (upper panel). RT-PCR assays showing specific association of several transcripts with CUGBP1 immunoprecipitates. *Gapdh*, *cMyc* and *Plk2* are negative controls. (**B**) Schematic of ribonucleoprotein immunoprecipitation microarray (RIP-Chip) experiment. The signal-to-negative ratio is the mean of probe set values of immunoprecipitated samples (α-CUGBP1) to that of negative control samples (control IgG). (**C**) Distribution of signal-to-negative ratios (see Dataset S2 for the complete list). The 95^th^-percentile value (indicated in the graph) was used as the cut-off for mRNAs showing a positive association with CUGBP1. The ratios for transcripts assayed by RT-PCR in (A) and Figure S3 are shown as dots with red and green colors representing positive and negative immunoprecipitation, respectively.

The results of our half life analysis indicated that labile mRNAs in myoblasts are more likely to contain GREs. These elements are known to recruit the RNA-binding protein CUGBP1 in T-cells and in other systems [21], [31]. It was therefore of interest to determine whether the labile GRE-containing transcripts in muscle might be targets of CUGBP1. In order to isolate mRNAs associated with CUGBP1 we prepared cytoplasmic lysates from C2C12 myoblasts and used a monoclonal anti-CUGBP1 antibody to immunoprecipitate the protein along with any associated mRNAs. Normal mouse IgG was used as a negative control. We then isolated RNA from both the CUGBP1 and control immunoprecipitates and determined whether specific GRE-containing, unstable transcripts were present by RT-PCR. We also looked for unstable transcripts that did not contain GREs, and for long-lived transcripts. As shown in Figure 4A, three GRE-containing mRNAs *Jun* (t_½_ = 43 min), *Smad7* (t_½_ = 61 min) and *Rnd3* (t_½_ = 34 min) were all enriched in the RNA pulled down with CUGBP1 compared with the control IgG. The *Jun* mRNA has been shown previously to interact with the *Xenopus* homolog of CUGBP1 (EDEN-BP; [32]) but the finding that CUGBP1 interacts with *Smad7* and *Rnd3* mRNAs is novel and quite intriguing as the proteins encoded by both these transcripts have been linked with the differentiation process in muscle [33], [34]. In contrast, the abundant and comparatively stable *Gapdh* mRNA was not present in either the control or CUGBP1 precipitated RNA samples. Finally, two transcripts with short half lives but lacking obvious GREs, *Myc* (t_½_ = 30 min) and *Plk2* (t_½_ = 34 min), also failed to show enrichment in the CUGBP1 immunoprecipitation (Figure 4A).

### Global identification of mRNAs associated with CUGBP1

We were encouraged by the discovery that CUGBP1 associates with certain GRE-containing mRNAs in C2C12 cells and therefore wanted to identify the full complement of mRNAs bound. The same basic immunoprecipitation protocol was used but following isolation from the immunoprecipitate, the RNA samples from the control IgG and anti-CUGBP1 precipitate were utilized to prepare DNA probes for hybridization to Affymetrix Mouse Gene 1.0 arrays (RIP-Chip [35]). The data were normalized and a ratio of the signal in the CUGBP1 immunoprecipitated sample compared to the negative control sample was calculated for each mRNA (signal-to-negative; Dataset S2). The procedure is summarized in Figure 4B. Transcripts were ranked by ratio and the top 5% (881 mRNAs) were designated as being associated with CUGBP1. These mRNAs are enriched from 3 to 23 fold in the CUGBP1 immunoprecipitate as compared to the control. As seen in Figure 4C, the mRNAs identified above as being bound by CUGBP1 fell within this set (red dots), while those that did not bind fell outside (green dots). Moreover, many transcripts previously identified by others as substrates for EDEN-BP (the *Xenopus* homolog of CUGBP1) were also found in the CUGBP1 immunoprecipitate including *Lmo4*, *Jun*, *Npm1*, *Aurka*, *Aurkb*, *Wee1* and *Bub3* [22]. *Lmo4* has also been previously identified as a substrate of CUGBP1 in mammalian cells [36]. The association of several additional transcripts with CUGBP1 was validated by RT-PCR (Figure S3).

**Figure 5:**
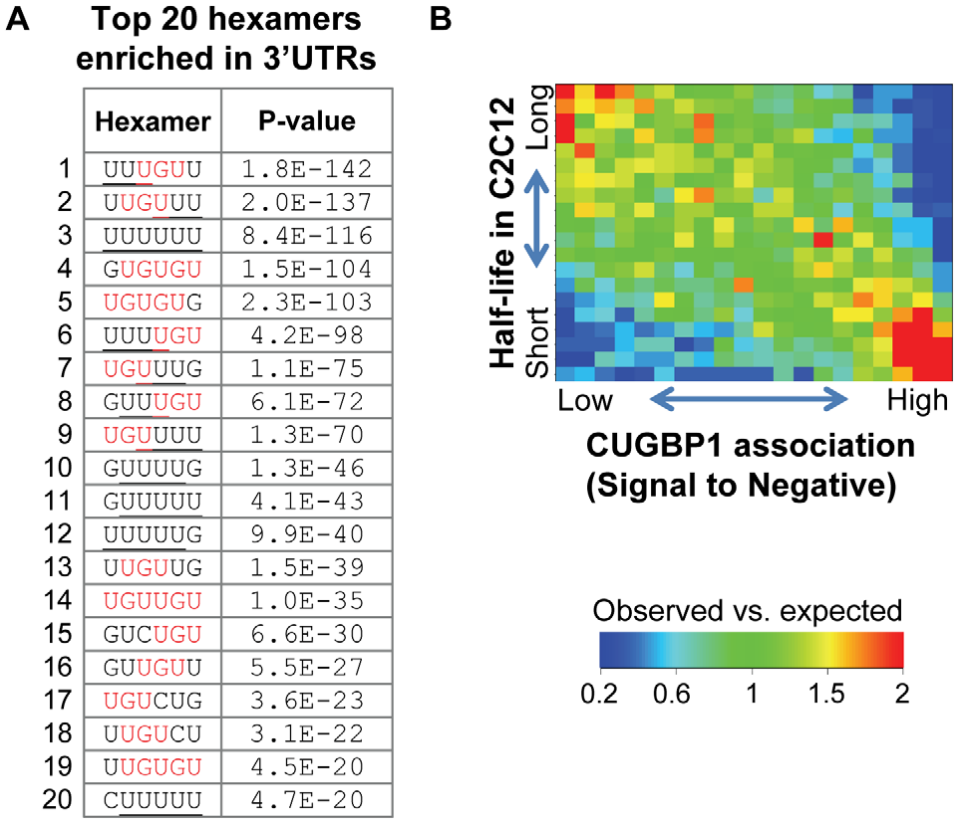
CUGBP1 bound mRNAs contain GREs in their 3′UTRs. (**A**) Top 20 ranked significant hexamers in the 3′UTRs of mRNAs with signal-to-negative ratios above 95^th^-percentile. P-values were derived from Fisher's exact test comparing frequency of occurrence in top 5% transcripts with that in other transcripts. Consecutive Us > = 3 are underlined, and UGU is shown in red. (**B**) Comparison of signal-to-negative ratios in the CUGBP1 RIP-Chip experiment with mRNA half lives in C2C12 cells. A gene density plot was used to show the relationship, in which mRNAs were evenly divided into 20 groups based on signal-to-negative ratio (x-axis) or half life (y-axis). The number of mRNAs in each cell of the 20×20 table (observed value, obs) was normalized to the mean of all cells (expected value). The ratios (observed/expected) are shown in a heat map according to the color scale shown in the figure. Red represents enrichment, and Blue for depletion.

Although CUGBP1 binds to pre-mRNAs to regulate splice site choice [37], we did not expect to see appreciable enrichment of these targets in our immunoprecipitate as at steady state pre-mRNA is generally a relatively small proportion of the total mRNA produced from each gene. In addition, we used cytoplasmic lysates which should not contain significant amounts of pre-mRNA. Thus, we expected the majority of CUGBP1 bound mRNAs to interact with CUGBP1 through their UTRs. As mRNA stability factors, including CUGBP1, predominantly interact with 3′UTRs we focused on these. By comparing the 3′UTR sequences of genes scoring in the top 5% of signal-to-negative ratios, we were able to discern a very significant enrichment of GU-rich hexamers in the immunoprecipitated mRNAs (Figure 5A and Table S1), including U-rich (≥3 consecutive Us), UGU flanked by Us, and UGUG/GUGU hexamers. These sequences closely resemble the binding site of CUGBP1 as characterized by SELEX and 3-hybrid analyses [31], [38]. Importantly, the GREs enriched in the CUGBP1 immunoprecipitate are also very similar to those enriched in the set of unstable mRNAs and include both UGUGU (DE4-like) and UUGUU (DE3-like) elements. Moreover, the association with short half life transcripts is clearly sequence specific as AREs were not over-represented in the CUGBP1-bound mRNAs.

### Functional analysis of CUGBP1-associated mRNAs

Consistent with the observation that labile mRNAs were more likely to have GREs in their 3′UTRs, we found that CUGBP1 associated mRNAs generally had shorter half lives than those that were not immunoprecipitated. This can be visualized in the gene density plot shown in Figure 5B. In this plot, red indicates enrichment of genes and blue indicates depletion of genes. Thus, high signal-to-negative ratios (association with CUGBP1) correlate with short half lives while low signal-to-negative ratios (no association with CUGBP1) correlate with long half lives. This fits well with our knowledge of the role of CUGBP1 as a facilitator of mRNA decay [7], [21]. In addition, the CUGBP1-associated mRNAs, like the set of labile mRNAs, encode factors involved with cell cycle, but mRNAs whose functions are associated with RNA processing, intracellular transport, and apoptosis are also over-represented (Table 2).

**Table 2:** Top ranked Gene Ontology (GO) terms associated with mRNAs immunoprecipitated with CUGBP1.

P-value^1^	GO ID, GO Term
5.60E-15	GO:0007049, cell cycle
5.07E-13	GO:0046907, intracellular transport
6.51E-13	GO:0008104, protein localization
1.11E-12	GO:0051641, cellular localization
7.98E-11	GO:0048522, positive regulation of cellular process
1.01E-10	GO:0048523, negative regulation of cellular process
1.46E-10	GO:0050793, regulation of developmental process
1.02E-09	GO:0006996, organelle organization
3.66E-09	GO:0009887, organ morphogenesis
7.94E-09	GO:0007242, intracellular signaling cascade
1.72E-08	GO:0006915, apoptosis
1.05E-07	GO:0000278, mitotic cell cycle
1.31E-07	GO:0009790, embryonic development
2.20E-07	GO:0006396, RNA processing
2.22E-07	GO:0016192, vesicle-mediated transport
4.20E-07	GO:0040007, growth
6.54E-07	GO:0065008, regulation of biological quality
1.64E-06	GO:0008283, cell proliferation
3.66E-06	GO:0000087, M phase of mitotic cell cycle
1.73E-05	GO:0000279, M phase

1 P-values were derived from Fisher's exact test, which indicates significance of enrichment of GO terms associated with top 5% of all mRNAs based on the signal-to-negative ratio. Top 20 ranked GO entries are shown.

### A subset of CUGBP1 targets are shared with HuR and/or Pum1

The sequence preferences of CUGBP1 overlap with those of HuR and Pum1 proteins, both of which are also able to bind to elements with a core UGU trinucleotide. The preferred binding site of HuR was recently defined as UUU(G/U)UUU [24] and that of Pum1 is UGUANAUA [39], [40]. We therefore compared the set of mRNAs that immunoprecipitated with CUGBP1 with similar sets generated for HuR ([41]) and Pum1 ([39], [40]). These data sets were generated using human cell lines under various conditions but we were nevertheless able to uncover significant overlap between them (Table S3). Indeed over 50 transcripts are bound by all three of these RNA-binding proteins. The transcripts shared by HuR and CUGBP1 are enriched in factors linked with cell cycle and post-transcriptional regulation of gene expression, while those shared by CUGBP1 and Pum1 are associated with cell proliferation (Table S4). Interestingly, CUGBP1 and HuR can be co-immunoprecipitated in an RNA-dependent manner, supporting the existence of mRNPs containing both CUGBP1 and HuR (data not shown). However, further work will be required to determine whether competition or co-association occurs during binding of these RBPs to individual transcripts.

### Depletion of CUGBP1 results in stabilization of target transcripts

**Figure 6:**
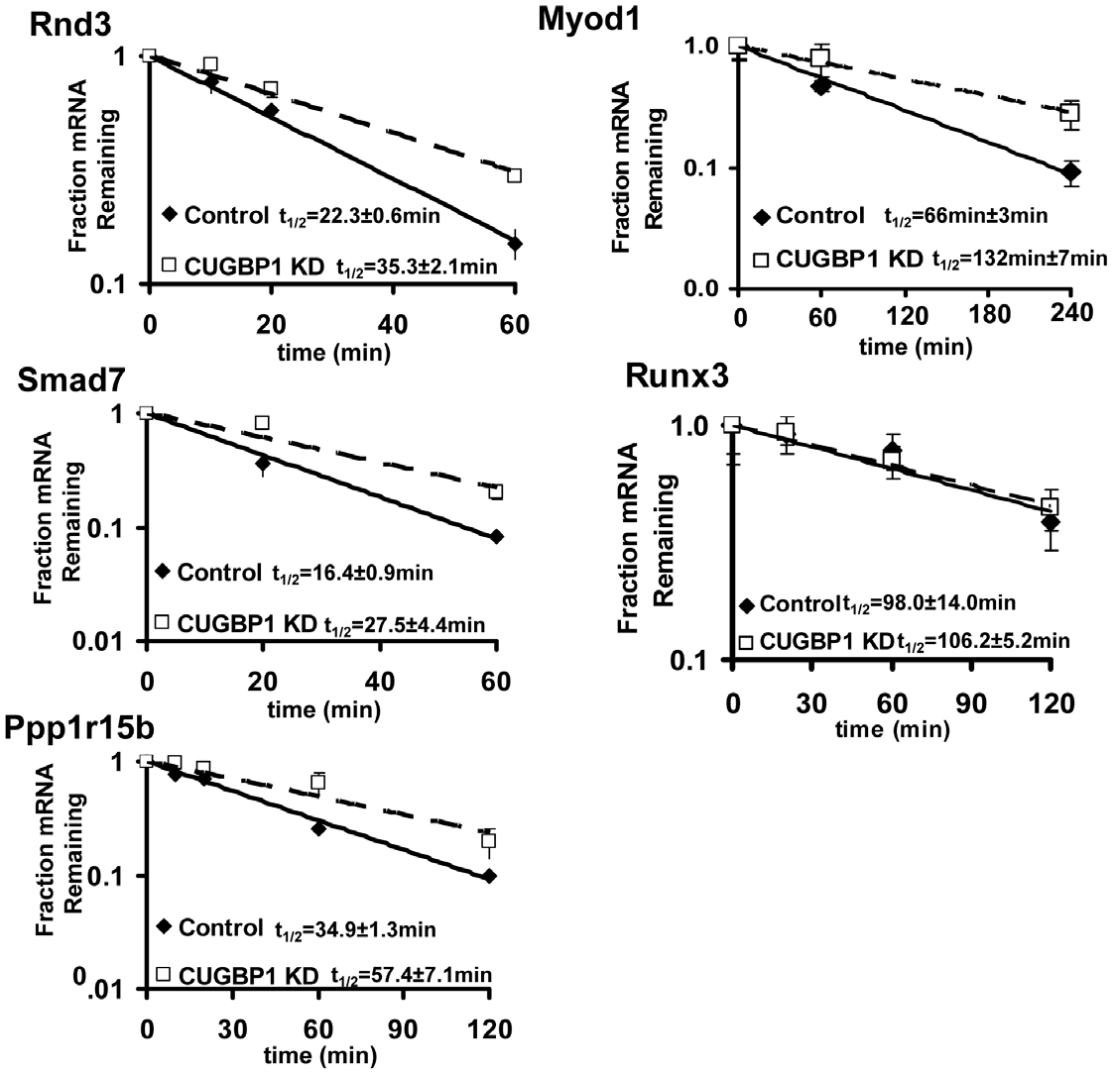
CUGBP1 bound mRNAs are stabilized in CUGBP1 knockdown cells. The decay rates of the indicated mRNAs were assessed in C2C12 (LKO1) and CUGBP1 KD cells by qRT-PCR following transcription inhibition with actinomycin D. Abundance of the mRNA of interest was normalized to *Gapdh* at each time point. Each half life was measured three times in each cell line. Representative results are depicted.

Finally, given the strong evidence of CUGBP1 binding to GREs in short-lived mRNAs we wished to determine whether CUGBP1 directly influences mRNA decay rates. We utilized a previously described C2C12 line (CUGBP1 KD) in which CUGBP1 is stably knocked down through expression of an shRNA targeting the 3′UTR [15]. We examined changes in mRNA decay rates in the CUGBP1 KD cell line following inhibition of transcription with Actinomycin D. The abundance of transcripts of interest was quantified by qRT-PCR and normalized to *Gapdh* which does not decay significantly over the time course used. We chose to examine five transcripts that immunoprecipitated with CUGBP1 (*Ppp1r15b*, *Rnd3*, *Smad7*, *Myod1* and *Runx3*). Of these mRNAs, which were enriched between 4.4 and 13.7 fold in the CUGBP1 immunoprecipitate, the first four showed significant stabilization in the KD cell line while *Runx3* mRNA was not affected (Figure 6). Interestingly, examination of the 3′UTRs of these five transcripts reveals that all have strong matches to GREs. *Runx3* was the most stable mRNA we looked at (t_1/2_∼98min) and has extensive CU-rich and CA-rich elements in its 3′UTR that resemble those we identified as stabilizing elements. We suggest that these may over-ride any instability mediated by CUGBP1 under the conditions used here. We find it particularly exciting that CUGBP1 KD stabilizes the *Myod1* mRNA as this transcript which encodes a muscle specific transcription factor exhibits dramatic stabilization during myogenesis through association of the HuR protein.

## Discussion

In this study we have determined decay rates for over 7,000 mRNAs in mouse C2C12 myoblasts. We identified GO terms associated with stable and unstable mRNAs and discovered specific 3′UTR sequence elements associated with instability (GREs and AREs) and with stability (CA, CU and GA-rich elements). Our analysis revealed that specific elements impact mRNA decay rates to different extents in C2C12 and ES cells. We also demonstrated that CUGBP1 associates with GRE-containing mRNAs in C2C12s and that some of these substrates are shared with Pum1 and/or HuR. Finally, knockdown of CUGBP1 results in stabilization of several GRE-containing mRNAs. These data demonstrate a clear role for CUGBP1 as an important regulator of mRNA decay in muscle cells.

### Cell-type specific regulation of mRNA decay rates

Comparison of our half lives to those generated by a similar approach in pluripotent and differentiating ES cells showed a good general correlation (Figure 3A), but some differences are evident. First, our median half life (2.9hr) is significantly shorter than those determined previously for ES cells (+LIF = 7.1 hr, −LIF = 5.5hr, +RA = 8.6hr [10]) and other cell types (HepG2>10hr [42], NIH3T3 = 4.6hr [4]). Although this difference could be authentic, we designed our experiment to favor accurate determination of short half lives which may have resulted in longer half lives being under-represented as they were less likely to meet our stringent criteria. Similarly, the ES cell experiment [10] favored longer half lives as the first time point was taken at 1 hr, thus the median half lives in these cases may be an overestimate.

We were interested to note that the GO terms most significantly associated with instability vary between cell types. Although mRNAs encoding factors linked with transcription regulation are unstable in all cell types examined to date (Figure S2B) [10], [42], [43], cell cycle associated mRNAs are unstable in C2C12 and HepG2 [42], but not in ES cells [10], human B cells [43] or NIH3T3 fibroblasts [43]. Intriguingly, mRNAs encoding factors associated with RNA processing functions appear to be exclusively destabilized in C2C12s as this GO term is not significantly linked with instability in other cell types [10], [42], [43].

There are also clear differences in half life between cell types for certain sets of genes suggesting that changes in mRNA decay rates may make very significant contributions to coordination of gene expression during differentiation. For example, ES(−LIF) cells show elevated stability for mRNAs coding for proteins involved in urogenital system and skeletal development, while in ES (+RA) cells mRNAs encoding factors linked with “neurogenesis” and “localization of cell” are stabilized (Figure S2). Since removal of LIF induces endodermal differentiation [44] and addition of RA favors neuronal differentiation [45], these results are consistent with selective stabilization of mRNAs in certain functional groups to coordinate differentiation programs.

### Impact of GREs and AREs on mRNA stability is also cell-type specific

We discovered that in C2C12s, GU-rich and AU-rich elements are over-represented in the 3′UTRs of unstable transcripts. Global analyses of mRNA decay in several cell types have reproducibly linked AREs with instability [1], [10], [42]. However, our results suggest that the impact of GREs may vary in different cell types. For example, in primary human T-cells GREs of the type UGUUUGUUUGU (related to DE3) were enriched in short lived mRNAs, but other GREs such as UGUGU (DE4) were not identified [21]. In HepG2 and Bud8 cells, there was strong correlation of UUUUUUU with instability, exactly as we found in C2C12s, but no evidence for any impact of GREs on mRNA half life [42]. Finally, when we investigated the influence of our destabilizing elements on decay rates in ES cells, we found that both GREs and AREs correlate with instability in all three conditions but AREs (especially non-AUUUA type) seem to be more significant in ES cells and their derivatives than in C2C12s. Notably, DE4 type (UGUGU) elements impact decay more in C2C12s than in ES cells (Figure 3B). We conclude that certain elements likely contribute to regulating gene expression patterns in a cell type-specific manner. The fact that UGUGU elements impact mRNA decay in myoblasts more than other cell types could be due to muscle-specific mRNAs being more likely to contain these elements, and/or to a specific factor such as CUGBP1 being more active in myoblasts.

### CUGBP1 regulons

A close examination of the mRNAs pulled down in the RIP-Chip experiment (Dataset S2) reveals that there are classes of transcripts with linked roles in cellular metabolism whose expression may be coordinately controlled through CUGBP1 association. For example, mRNAs encoding three out of six protein subunits of the Signal Recognition Particle (*Srp54b*, *Srp68*, *Srp72*), which recognizes the signal peptide of membrane and secretory proteins and directs their translocation into the endoplasmic reticulum (ER) [46], are in the set of CUGBP1-bound transcripts. All three contain strong GREs in their 3′UTRs. Overall mRNAs encoding factors associated with the GO cellular component “endoplasmic reticulum” are significantly enriched in the set of immunoprecipitated transcripts (p = 2.34×10^−6^). In particular, several transcripts encoding factors required for later steps in entry and processing of proteins in the ER are also immunoprecipitated by CUGBP1 (e.g. Translocon components (*Tram1*,*Ssr1*) Signal peptidases (*Spcs2*, *Sppl3*), Oligosaccharyltransferase subunits (*Stt3a*, *Dad1 Krtcap2*) and Chaperones (*Calr*, *Canx*)). We therefore suggest that CUGBP1 may be an important regulator of ER homeostasis. In another putative RNA regulon, mRNAs encoding three components of the telomeric Shelterin Complex (*Terf1*, *Terf2*, *Pot1a*) [47] are also present in CUGBP1 immunoprecipitates.

### Networked regulation of RNA-binding proteins

A recent study of six RNA-binding proteins (AUF1, HuR(ElavL1), NF90, TIA-1, TIAR, and KSRP) found that each of these factors bound to its own mRNA and that each also regulated expression of one or more of the others [48]. We can now add CUGBP1 to this class of RNA-binding proteins that has the capacity to autoregulate, as we found CUGBP1 bound to its own mRNA. Moreover, we found CUGBP1 associates with the transcripts encoding a plethora of RNA-binding proteins including several splicing factors as well as PABPN1, PABPC4, HuR(ElavL1) and Pum1 (Dataset S2). This lends additional support to recent studies suggesting that a complex network of self and cross-regulation exists for RNA-binding proteins involved in post-transcriptional control [48].

### Competition and/or cooperation with other RBPs

Many of the transcripts we found associated with CUGBP1 have also been pulled down in RIP-Chip experiments performed in human cells using anti-HuR and/or anti-Pumilio (Pum1) antibodies [39]–[41] (Table S3). One question that arises is whether CUGBP1 and these other RNA-binding factors (both of which also regulate mRNA decay and translation) are binding at separate sites on the same transcript or competing for the same binding site. HuR has been shown to compete with another CELF family member, CUGBP2, for binding to the *Ptgs2(Cox2)* 3′UTR to regulate translation [49]. Similar competition could easily occur with CUGBP1, which is very homologous to CUGBP2, as *Ptgs2* mRNA was immunoprecipitated in our RIP-Chip. Competition between HuR and CUGBP1 for binding to RNA targets could be a relatively common event as there are clear similarities between the most recently defined U-rich binding preferences of HuR [24] and the CUGBP1 binding elements defined here and by others [31], [38]. In *Xenopus* embryos adjacent Pum1 and CUGBP1 binding sites are necessary to achieve spatially regulated translation of the xCR1 mRNA [50]. Pum1 binding sites may also overlap with CUGBP1 sites, as both proteins have preferences for U/purine rich sequences.

As CUGBP1 plays such an important role in muscle cells, we looked more closely at CUGBP1-associated transcripts that encode proteins implicated in the myogenesis process. Of note, *Myod1* [14], *Myog* [14], *Cdkn1a* [14], *Eif4e* [51], *Ccnd1* [52], and *Dusp1(Mkp1)* [53] mRNAs can all be recognized by both CUGBP1 and HuR and exhibit regulated decay and/or translation. Importantly, the first four of these mRNAs are significantly up-regulated during myogenic differentiation [51], [54]. Several other factors encoded by CUGBP1 bound mRNAs are also up-regulated during myogenesis, including *Eif4ebp1* [54], *CD9* [55], *Cdon* [56], *Igfbp5* [57], *Smad7* [33] and *Rnd3* [34]. In Figure 6, we have shown that *Myod1* mRNA is stabilized in C2C12 myoblasts following CUGBP1 knockdown. We are currently investigating whether CUGBP1 coordinates with HuR to achieve appropriate regulation of myogenic factors such as *Myod1* upon cell cycle withdrawal and myoblast fusion during differentiation.

### Muscle-specific regulation by CUGBP1

CUGBP1 function and expression are altered dramatically in Type I myotonic dystrophy (DM1) [58]–[60]. There is also evidence that CUGBP1 is sequestered in Oculopharyngeal Muscular Dystrophy [61] and Fragile-X-Associated Tremor/Ataxia Syndrome [62] and over-expressed in Spinal Bulbar Muscular Atrophy [63]. In DM1, there is a great deal of compelling evidence for aberrant splice site selection in several clinically relevant transcripts [64]–[67] but fewer studies have examined effects on mRNA stability or translation. We have recently shown that mRNA stability may indeed be affected in DM1. A CUGBP1 target transcript, the TNF mRNA, is stabilized under DM-like conditions due to impaired CUGBP1 function [15]. The results presented here suggest that effects on mRNA stability may be a significant contributor to pathogenesis in neuromuscular diseases.

## Materials and Methods

### Cell culture

C2C12 cells were obtained from ATCC (CRL-1772). The two cell lines used here were described previously [15] and are stable lines derived from CRL-1772 containing either an empty lentivirus vector (LKO1) or a vector encoding an shRNA against CUGBP1. These cell lines were cultured in DMEM with 10% FBS, 10 U/ml penicillin, 10 µg/ml streptomycin, and 1µg/ml puromycin. The cells were maintained below 70% confluency to prevent differentiation.

### mRNA half life sample collection and quality control

C2C12(LKO1) cells were grown to <70% confluency, transcription was inhibited by addition of Actinomycin-D (8µg/mL Sigma) for a period of 30 min. Cells were collected in Trizol™ (Invitrogen) at the indicated time points following the incubation and total RNA was prepared according to the manufacturers instructions. RNA concentration, purity, and quality were determined via Bioanalyzer (Agilent). Transcription shut-off was verified using 1µg of total RNA in qRT-PCR assays to measure half life of *Myod1* or *Myog* using *Gapdh* as a reference gene. For examination of changes in half life in CUGBP1 KD vs control(LKO1) cells, total RNA samples were collected from control and CUGBP1 KD cells and the abundance of the transcript of interest was determined by qRT-PCR in each time point using primers described in Table S5.

### RNA immunoprecipitation

Cell lysates were prepared from proliferating C2C12 (LKO-1) cells (<70% confluency) as previously described [68]. Immunoprecipitates were isolated by incubating 100µl of cleared lysate with 7µl of normal IgG (Santa Cruz sc-3877) or αCUGBP1 antibody (monoclonal 3B1) for 1 hour on ice. Following brief centrifugation at 4°C, the reaction was transferred to 100µl of a 10% slurry of Protein-G sepharose beads (Sigma) in NT-2 buffer (50 mM Tris pH7.4, 150 mM NaCl, 1 mM MgCl_2_, 0.05% Nonidet P-40) and rocked for 1 hour at 4°C. Beads were washed twice with 250µl of NT-2 buffer, transferred to a micro-spin column (Pierce) and washed four more times with 200µL of NT-2 buffer. Beads were collected and presence of RNA or proteins was assessed by RT-PCR or Western Blot respectively. For RNA analysis, Trizol™ (Invitrogen) was added to beads for elution, and RNA isolated according to the manufacturer's instructions. 1 µl of RNA was reverse-transcribed with random hexamers, and the resulting cDNA was used in a PCR reaction to amplify the gene of interest. Reaction products were separated on a 2% agarose gel stained with ethidium bromide. Proteins were eluted with 6× SDS loading buffer and boiled for 5 minutes before being resolved by SDS-PAGE (10% gels) and analyzed by western blot.

### Preparation of RNA samples and microarray hybridization

RNA samples were isolated from Trizol™ (Invitrogen) following the manufacturer's protocol. 300ng of total RNA for half life arrays, or 100ng for immunoprecipitation arrays, were used to generate labeled cDNA fragments for hybridization to Mouse Affymetrix Gene 1.0 ST Arrays following the manufacturer's protocol (GeneChip™ WT cDNA Synthesis Kit #900652 and #900720). Production of probes and hybridization was performed by the Colorado State University Genomics and Proteomics Core Facility. Half life experiments were conducted in triplicate, with each time point hybridized to a single array. Immunoprecipitations were performed in duplicate for input, CUGBP1 immunoprecipitated (3B1), and normal mouse IgG (Santa Cruz sc-3877) immunoprecipitated RNAs, with each sample being hybridized to a single array. The signal-to-negative ratio given in Dataset S2 is the mean of probe set values of immunoprecipitated samples (α-CUGBP1) to that of negative control samples (control IgG).

### Microarray analysis

Total RNA was prepared from each sample and used to generate probes for hybridization to Affymetrix Mouse Gene 1.0 arrays. Microarray analysis was performed on samples collected 0, 10 min, 50 min, 110 min and 230 min after treatment with Actinomycin D. Transcripts whose probe sets with detection above background P-value <0.05 in at least 2 out of 3 replicates at the 0 min time point were considered expressed and used for subsequent analyses. All probe set values were normalized to the 5^th^ percentile value of all probe sets on the same array. All data is MIAME compliant and the raw data has been deposited in the GEO database (accession # GSE21236).

### Half life calculations

A nonlinear least squares model, as described in [69], was used to calculate half lives using the microarray data. A transcript was considered to have reliable half life measurement if 1) the microarray data had a good fit to the nonlinear least squares model (P-value<0.05) and 2) the 95% confidence interval for half life is less than two times the half life. Transcripts with reliable half lives in at least two of three replicates were selected for further analyses.

### GO analysis

To identify gene functional groups that have significantly biased half lives, we examined Gene Ontology (GO) terms for the genes encoding most stable 10% and least stable 10% of transcripts. Fisher's exact test was performed to identify significantly enriched GO terms for selected genes. Similar methods were used to identify significant GO terms for transcripts co-immunoprecipitated with CUGBP1, and transcripts that bind CUGBP1 and/or Pum1 and HuR. For comparison between cell types, we used only genes that were expressed and had good half life measurements in all cells used in this study.

### *Cis* element analysis

To identify destabilizing and stabilizing elements in 3′UTRs, we took the most stable 10% and least stable 10% of transcripts and examined hexamers in their 3′UTRs. The significance of overrepresentation of a hexamer in each set was calculated by the Fisher's exact test comparing the two sets. Similar methods were used to obtain significance of overrepresentation for hexamers in 3′UTRs of transcripts co-immunoprecipitated with CUGBP1. The 3′UTR sequences were obtained from the UCSC RefSeq database. Hexamers were clustered to make *cis* element motifs as described in [25]. Briefly, selected significant hexamers were compared based on their sequence similarities. Similar hexamers were grouped to make position-specific scoring matrices (PSSMs) and sequence logos. PSSMs were used to scan sequences for matches with the *cis* elements. For comparison between cell types, we used only genes that were expressed and had good half life measurements in all cells used in this study.

## Supporting Information

Dataset S1 Half lives for 7398 mRNAs in C2C12 (LKO1) cells.
(1.87 MB XLS)

Dataset S2 Signal-to-negative ratio for mRNAs immunoprecipitated with CUGBP1.
(1.98 MB XLS)

Figure S1 Clustering of hexamers into cis-element groups for motif building(see Materials and Methods for details).
(1.60 MB TIF)

Figure S2 Difference in mRNA half life across cells types. (A) Ratios of half lives in ES, ES/LIF-, and ES/RA+ cells to those in C2C12 were calculated, normalized by row mean, and presented in a heat map according to the color scale show at the bottom of the graph. (B) Significant Gene Ontology (GO) terms associated with mRNAs with short and long half lives in different cell types. Significance scores (SS) were calculated for each GO term. SS = −log(P-value)*s, where P-value was based on Kolmogorov-Smirnov Tests, and s was 1 if a GO term was more significantly associated with mRNAs with long half lives or −1 otherwise. SS are shown in a heatmap according to the color scale show in the figure. Only those GO terms with SS >3 or <−3 in at least one cell type are shown.
(0.68 MB TIF)

Figure S3 Validation of RIP-Chip results by RT-PCR. RNAs immunoprecipitated by anti-CUGBP1 or normal IgG were subject to RT-PCR with primers specific to the indicated genes. PCR products were visualized on a 2% agarose gel stained with ethidium bromide. Input lanes contain 10% of the RNA isolated from samples prior to immunoprecipitation.
(0.22 MB TIF)

Table S1 Hexamer scores for half life and RIP-Chip analyses. For the half life analysis, association of each hexamer with short and long half life mRNAs was assessed and a score derived using the equation Significance Score = −log(P-value)*s, where P value was based on Fisher's exact test and s = 1 if the hexamer is over-represented in long half life mRNAs or −1 if it is over-represented in short half life mRNAs. For the RIP-Chip analysis, the occurrence of each hexamer within the set of mRNAs immunoprecipitated with CUGBP1 was assessed. The value shown is −log(P-value) where P-value was based on Fisher's exact test.
(0.57 MB XLS)

Table S2 Contribution of cis-elements in different regions of the mRNA to half life, analyzed by a linear model. The linear model is based on y = a + b1x1 + b2x2 + b3x3 … + bnxn, where y is mRNA half life, a is intercept of the model, x1…xn are different features of mRNA, including scores of DEs and SEs in different regions of mRNA (3′UTR, CDS, or 5′UTR) and size of each region, and b1…bn are slopes for x1…xn. Slope >0 indicates contribution to stabilization and slope<0 indicates contribution to destabilization. The P-value for bn indicates its significance, i.e. probability that bn is 0 (null hypothesis). Size is sequence size.
(0.05 MB DOC)

Table S3 Lists of mRNA targets shared by CUGBP1, HuR and/or Pum1. Datasets from RIP Chip experiments using HuR and Pum1 antibodies [39]–[41] were compared with the set of mRNAs immunoprecipitated with CUGBP1 using Ingenuity Pathways Analysis (Ingenuity Systems, www.ingenuity.com). Symbols of genes whose mRNAs are bound by the indicated RNA-binding proteins are listed.
(0.03 MB XLS)

Table S4 Top-ranked Gene Ontology Terms associated with shared target mRNAs of CUGBP1, HuR and Pum1. P-values were derived from Fisher's exact test, which indicates significance of enrichment of GO terms. The top six ranked GO terms are shown.
(0.05 MB DOC)

Table S5 Primers used in qRT-PCR and RT-PCR analysis.
(0.02 MB XLS)
