# Supplementary figures and images for: Systematic Analysis of Cis-Elements in Unstable mRNAs Demonstrates that CUGBP1 Is a Key Regulator of mRNA Decay in Muscle Cells
Source: PLoS One. 2010 Jun 21;5(6):e11201. doi: 10.1371/journal.pone.0011201 (PMC2888570; doi:10.1371/journal.pone.0011201)

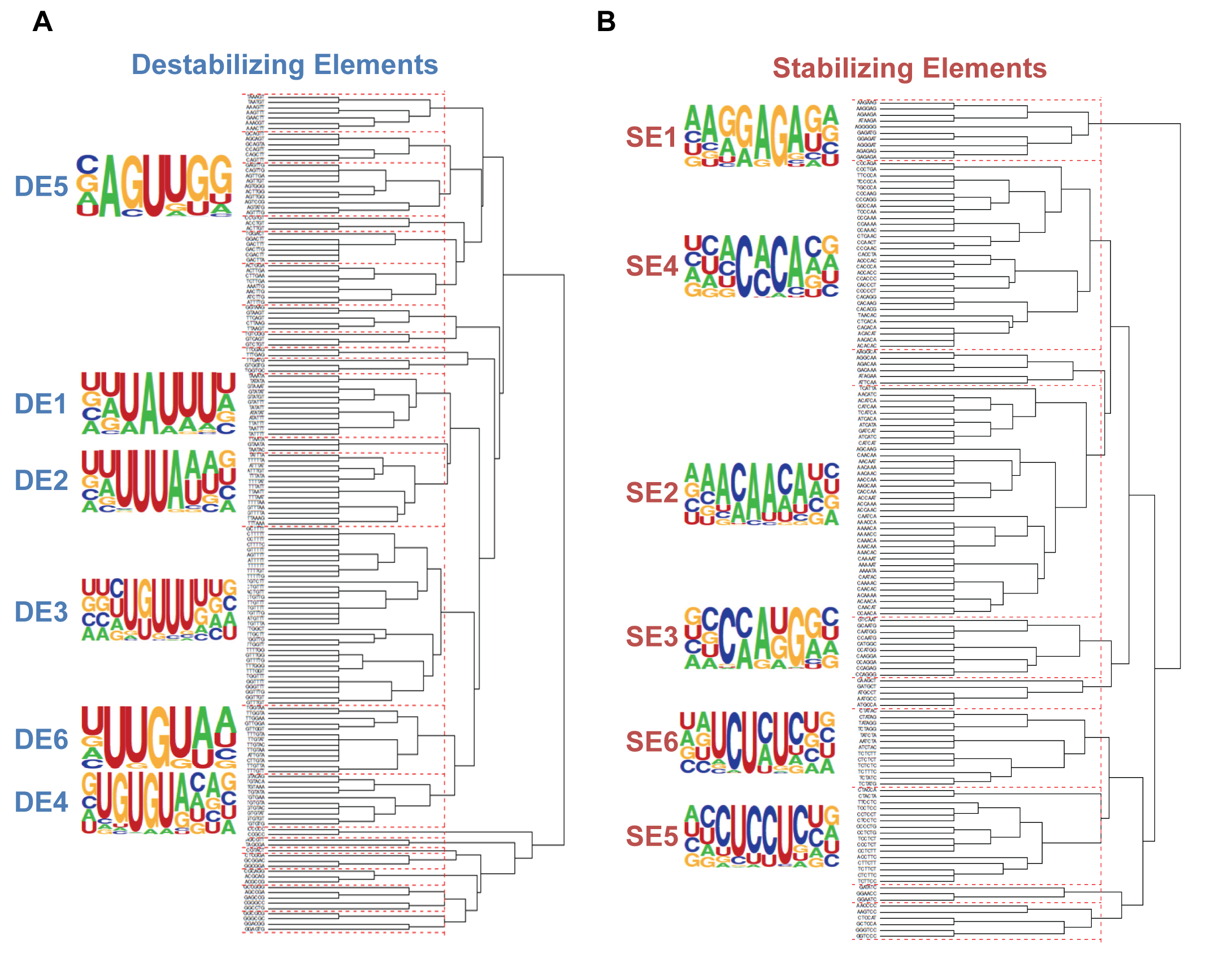

Supplement: Figure S1 — Clustering of hexamers into cis-element groups for motif building(see Materials and Methods for details). (1.60 MB TIF) [file pone.0011201.s003.tif]

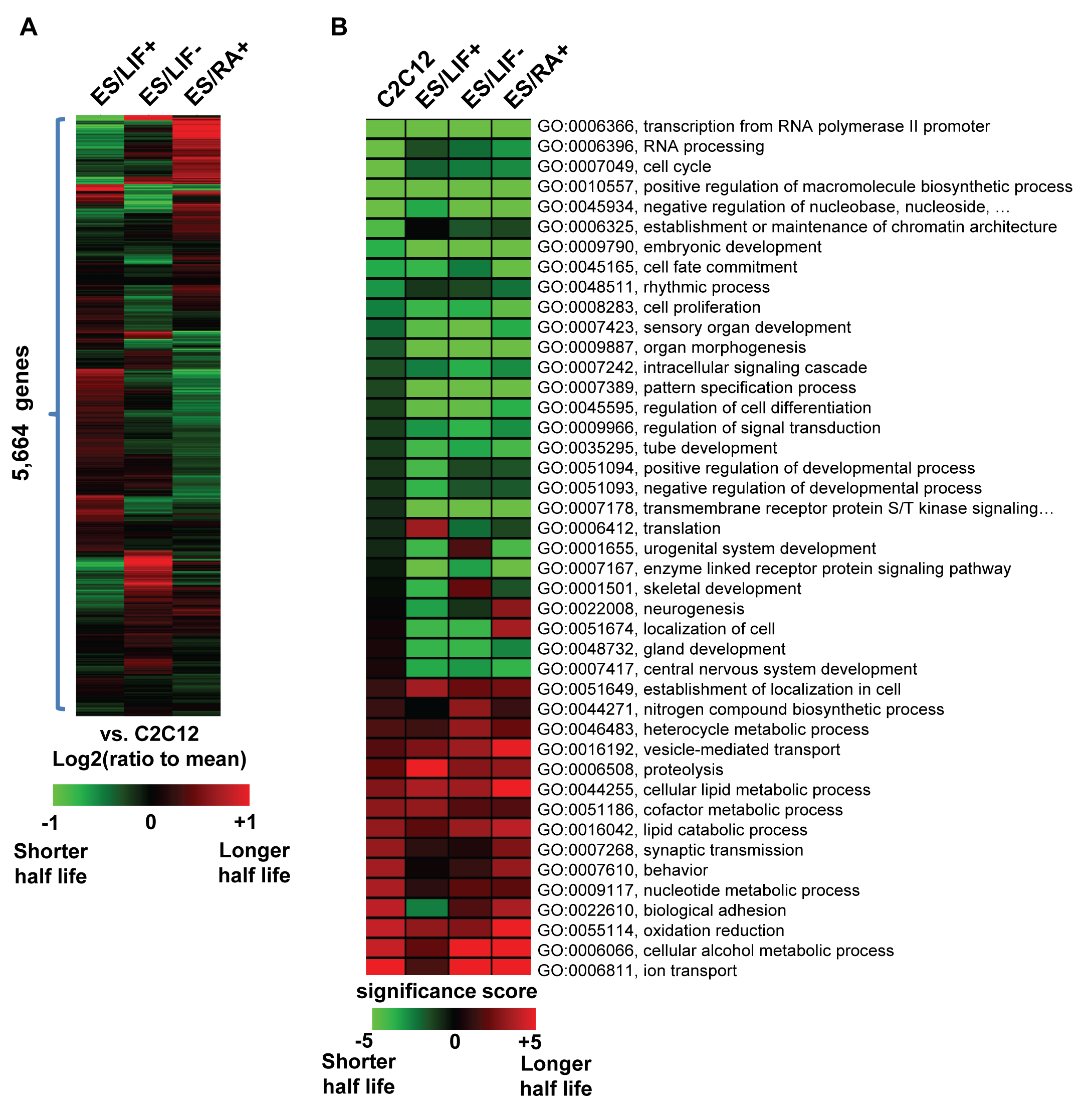

Supplement: Figure S2 — Difference in mRNA half life across cells types. (A) Ratios of half lives in ES, ES/LIF-, and ES/RA+ cells to those in C2C12 were calculated, normalized by row mean, and presented in a heat map according to the color scale show at the bottom of the graph. (B) Significant Gene Ontology (GO) terms associated with mRNAs with short and long half lives in different cell types. Significance scores (SS) were calculated for each GO term. SS = −log(P-value)*s, where P-value was based on Kolmogorov-Smirnov Tests, and s was 1 if a GO term was more significantly associated with mRNAs with long half lives or −1 otherwise. SS are shown in a heatmap according to the color scale show in the figure. Only those GO terms with SS >3 or <−3 in at least one cell type are shown. (0.68 MB TIF) [file pone.0011201.s004.tif]

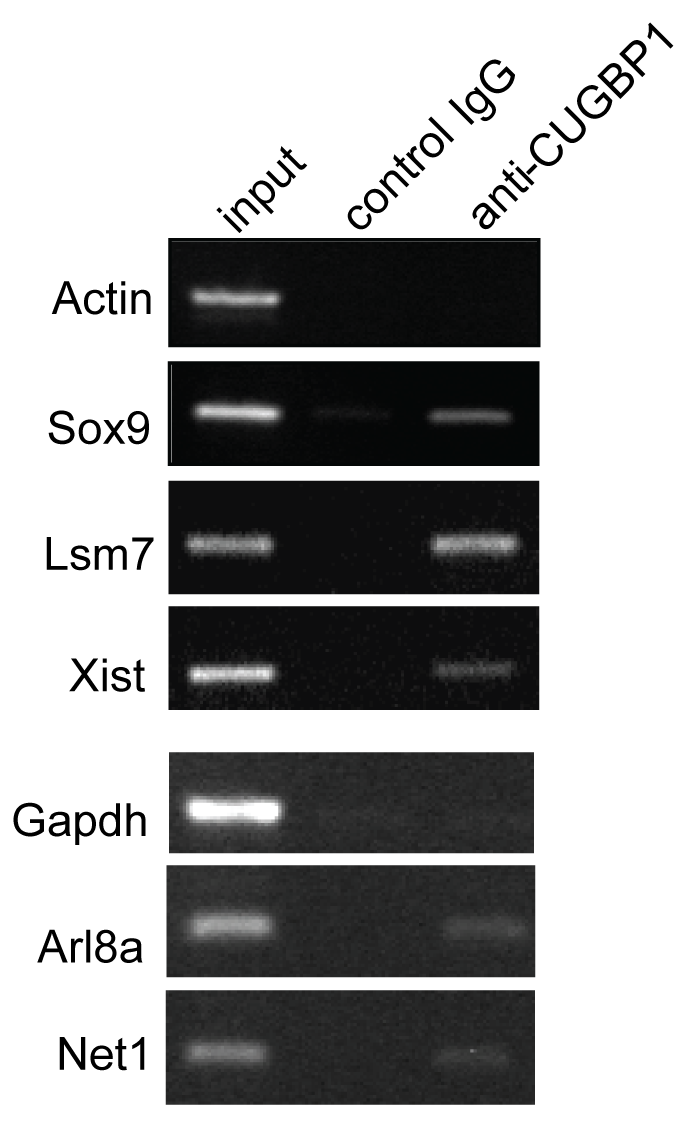

Supplement: Figure S3 — Validation of RIP-Chip results by RT-PCR. RNAs immunoprecipitated by anti-CUGBP1 or normal IgG were subject to RT-PCR with primers specific to the indicated genes. PCR products were visualized on a 2% agarose gel stained with ethidium bromide. Input lanes contain 10% of the RNA isolated from samples prior to immunoprecipitation. (0.22 MB TIF) [file pone.0011201.s005.tif]
