# Supplementary material for: Systematic Analysis of Cis-Elements in Unstable mRNAs Demonstrates that CUGBP1 Is a Key Regulator of mRNA Decay in Muscle Cells
Source: PLoS One. 2010 Jun 21;5(6):e11201. doi: 10.1371/journal.pone.0011201 (PMC2888570; doi:10.1371/journal.pone.0011201)
Supplement: Table S2 — Contribution of cis-elements in different regions of the mRNA to half life, analyzed by a linear model. The linear model is based on y = a + b1x1 + b2x2 + b3x3 … + bnxn, where y is mRNA half life, a is intercept of the model, x1…xn are different features of mRNA, including scores of DEs and SEs in different regions of mRNA (3′UTR, CDS, or 5′UTR) and size of each region, and b1…bn are slopes for x1…xn. Slope >0 indicates contribution to stabilization and slope<0 indicates contribution to destabilization. The P-value for bn indicates its significance, i.e. probability that bn is 0 (null hypothesis). Size is sequence size. (0.05 MB DOC) [file pone.0011201.s007.doc]

| Parameter | Slope | *P*-value | Slope | *P*-value | Slope | *P*-value |
| --- | --- | --- | --- | --- | --- | --- |
|  | 3’UTR | | CDS | | 5’UTR | |
| DE1 | 0.0002 | 9.9E-01 | -0.0050 | 6.2E-01 | -0.0083 | 3.5E-01 |
| DE2 | -0.0575 | 1.1E-06 | -0.0113 | 2.6E-01 | -0.0116 | 1.8E-01 |
| DE3 | -0.0456 | 3.7E-06 | -0.0030 | 7.8E-01 | -0.0261 | 4.8E-04 |
| DE4 | -0.0160 | 2.5E-01 | 0.0044 | 7.2E-01 | -0.0108 | 2.4E-01 |
| DE5 | -0.0266 | 9.8E-03 | -0.0054 | 6.6E-01 | -0.0109 | 9.4E-02 |
| DE6 | -0.0248 | 9.7E-03 | -0.0055 | 6.2E-01 | 0.0030 | 6.8E-01 |
| SE1 | 0.0019 | 8.7E-01 | -0.0306 | 7.4E-02 | 0.0041 | 6.3E-01 |
| SE2 | 0.0491 | 3.6E-04 | -0.0306 | 5.3E-02 | 0.0281 | 2.7E-03 |
| SE3 | 0.0386 | 3.6E-03 | 0.0434 | 1.2E-01 | 0.0119 | 1.8E-01 |
| SE4 | 0.0110 | 5.1E-01 | -0.0102 | 7.7E-01 | 0.0383 | 4.1E-05 |
| SE5 | 0.0309 | 1.1E-02 | 0.0213 | 1.4E-01 | 0.0004 | 9.6E-01 |
| SE6 | 0.0003 | 9.8E-01 | 0.0262 | 5.6E-02 | 0.0069 | 4.1E-01 |
| Size | 0 | 3.3E-02 | 0 | 6.9E-01 | -0.0003 | 7.0E-02 |
