# Supplementary material for: Systematic Analysis of Cis-Elements in Unstable mRNAs Demonstrates that CUGBP1 Is a Key Regulator of mRNA Decay in Muscle Cells
Source: PLoS One. 2010 Jun 21;5(6):e11201. doi: 10.1371/journal.pone.0011201 (PMC2888570; doi:10.1371/journal.pone.0011201)
Supplement: Table S4 — Top-ranked Gene Ontology Terms associated with shared target mRNAs of CUGBP1, HuR and Pum1. P-values were derived from Fisher's exact test, which indicates significance of enrichment of GO terms. The top six ranked GO terms are shown. (0.05 MB DOC) [file pone.0011201.s009.doc]

| P-value | GO ID, GO term |
| --- | --- |
| **Top-Ranked GO terms for transcripts bound by both HuR and CUGBP1** | |
| 9.67E-06 | GO:0007049, cell cycle |
| 1.25E-05 | GO:0022618, ribonucleoprotein complex assembly |
| 3.71E-05 | GO:0051246, regulation of protein metabolic process |
| 4.04E-05 | GO:0010608, posttranscriptional regulation of gene expression |
| 8.56E-05 | GO:0016071, mRNA metabolic process |
| 1.35E-04 | GO:0006397, mRNA processing |
| **Top-Ranked GO terms for transcripts bound by both Pum1 and CUGBP1** | |
| 2.20E-04 | GO:0042127, regulation of cell proliferation |
| 2.97E-04 | GO:0008283, cell proliferation |
| 3.63E-04 | GO:0008284, positive regulation of cell proliferation |
| 4.00E-04 | GO:0021532, neural tube patterning |
| 7.98E-04 | GO:0007049, cell cycle |
| 9.62E-04 | GO:0021915, neural tube development |
| **Top-Ranked GO terms for transcripts bound by both Pum1 and HuR** | |
| 3.96E-04 | GO:0007049, cell cycle |
| 1.56E-03 | GO:0008380, RNA splicing |
| 2.38E-03 | GO:0051301, cell division |
| 2.46E-03 | GO:0030835, negative regulation of actin filament depolymerization |
| 2.46E-03 | GO:0051693, actin filament capping |
| 3.36E-03 | GO:0030042, actin filament depolymerization |
| **Top-Ranked GO terms for transcripts bound by CUGBP1, Pum1 and HuR** | |
| 4.16E-03 | GO:0007049, cell cycle |
| 5.93E-03 | GO:0007264, small GTPase mediated signal transduction |
| 7.74E-03 | GO:0016055, Wnt receptor signaling pathway |
| 9.16E-03 | GO:0000279, M phase |
| 9.73E-03 | GO:0051246, regulation of protein metabolic process |
| 9.82E-03 | GO:0046578, regulation of Ras protein signal transduction |
